# Gene Co-Expression Network Analysis Unraveling Transcriptional Regulation of High-Altitude Adaptation of Tibetan Pig

**DOI:** 10.1371/journal.pone.0168161

**Published:** 2016-12-09

**Authors:** Cunling Jia, Xiaoyan Kong, James E. Koltes, Xiao Gou, Shuli Yang, Dawei Yan, Shaoxiong Lu, Zehui Wei

**Affiliations:** 1 College of Animal Science and Technology, Northwest A&F University, Yangling, Shaanxi, China; 2 College of Animal Science and Technology, Yunnan Agricultural University, Kunming, Yunnan, China; 3 Department of Animal Science, University of Arkansas, Fayetteville, AR, United States of America; Universitat de Lleida, SPAIN

## Abstract

Tibetan pigs have survived at high altitude for millennia and they have a suite of adaptive features to tolerate the hypoxic environment. However, the molecular mechanisms underlying the regulation of hypoxia-adaptive phenotypes have not been completely elucidated. In this study, we analyzed differentially expressed genes (DEGs), biological pathways and constructed co-expression regulation networks using whole-transcriptome microarrays from lung tissues of Tibetan and Duroc pigs both at high and low altitude. A total of 3,066 DEGs were identified and this list was over-represented for the ontology terms including metabolic process, catalytic activity, and KEGG pathway including metabolic pathway and PI3K-Akt signaling pathway. The regulatory (RIF) and phenotypic (PIF) impact factor analysis identified several known and several potentially novel regulators of hypoxia adaption, including: *IKBKG*, *KLF6* and *RBPJ* (RIF1), *SF3B1*, *EFEMP1*, *HOXB6* and *ATF6* (RIF2). These findings provide new details of the regulatory architecture of hypoxia-adaptive genes and also insight into which genes may undergo epigenetic modification for further study in the high-altitude adaptation.

## Introduction

The hypoxic environment at high altitude imposes extreme physiological challenges in mammals. Classical response to hypoxia is characterized by systemic changes in cardiovascular, respiratory, and hematopoietic functions that impact convective oxygen transport. Failure of these systems to adapt leads to altitude illness or even death [1, 2]. Native high-altitude species have been selected through evolutionary processes to have heritable genetic adaptations [3] in morphological, anatomical, physiological [4], biochemical, and behavioral traits to survive under low oxygen tension [5–7]. For example, Tibetan human populations showed lower hypoxic pulmonary vasoconstrictor response and higher blood oxygen saturation with similar hemoglobin levels to those expected at sea level [6–9]. Recently, a number of genome-wide analyses have been conducted to determine the genetic bases of adaptation to high altitude in species such as: highlander [10–15], yak [16, 17], Tibetan antelope [18], Tibetan mastiff [19], Tibetan pig [3, 20], Tibetan grey wolf [21], Tibetan Chicken [22], Tibetan cashmere goat [23] and Tibetan sheep [24]. Notable candidate genes related to high altitude adaptation included members of the HIF signaling pathway as well as genes associated with hemoglobin levels. However, the molecular mechanisms underlying adaptation to high altitude are yet to be completely elucidated.

The Diqing Tibetan pig (*Sus scrofa*) is a native breed that mainly inhabits Diqing Tibetan Autonomous Prefecture of the Yunnan province of China (average altitude: 3,700 m). Although their meat production, reproduction and growth rate is not as good as domesticated pigs living at sea level, adaptation to hypoxia, cold climate, disease and harsh environment makes the Tibetan pig the only adapted breed to these conditions. Tibetan pigs have adapted to high altitude with well-developed heart and lungs and a blunted erythropoietic response [20]. Since porcine physiology and anatomy are similar to humans [25], the Tibetan pig could be a suitable animal model for human high altitude diseases.

The objective of this study was to identify transcripts, pathways and potential regulators involved in high-altitude adaptation. Transcriptome profiling of lung tissues from Tibetan pigs and Duroc pigs was analyzed using Agilent microarrays, and potential pathway regulators were identified using two methods: partial correlation and information theory (PCIT) [26] and regulatory impact factor (RIF) algorithms [27–29]. The goal of this research is to identify pathways involved in high-altitude adaptation that may provide potential therapeutic solutions of hypoxia-induced diseases.

## Materials and Methods

### Ethics Statement

All animal experimental procedures were performed according to the Guide for the Care and Use of Laboratory Animals (Ministry of Science and Technology of China, 2006). The protocol was approved by the Experimental Animal Management Committee (EAMC) of Northwest A&F University ([2005]142).

### Animal and sample preparation

**Fig 1:**
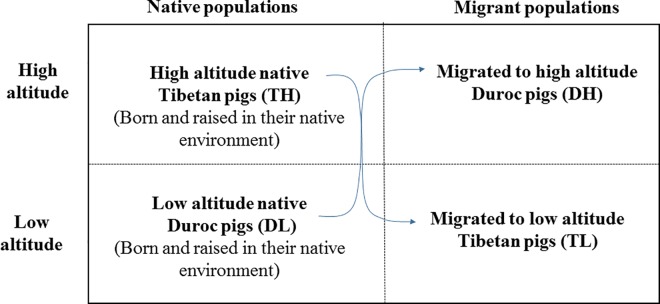
A migrant design to study the adaptation of Tibetan pigs to high altitude. The native population included Tibetan pigs living at high altitude (TH) and Duroc pigs living at low altitude (DL). At 1 month of age, half of the animals were migrated to low altitude (TL) or high altitude (DH). Animals were harvested at 6 month of age.

Tibetan pigs were raised at Yunnan (Shangri-La Specialized Farmers Cooperatives, Tibetan Autonomous Prefecture of Diqing, 3,500 m) (Tibetan pigs at high altitude, herein mentioned as TH), and Duroc pigs were raised at Guangdong (Guangdong Yifa Breeding Co. Ltd, Huizhou, 40 m) (Duroc pigs at low altitude, herein mentioned as DL). In our experiment, we employed a migrant study design. Sixteen Tibetan female piglets from the TH population and sixteen Duroc female piglets from the DL population were selected with similar weight and non-genetic relationship. Among these, 8 piglets from each groups were migrated to low altitude (TL) or high altitude (DH) from their original rearing facility at 1 month of age, respectively (Fig 1). Animal were fed and watered *ad libitum*. At 6 month of age, 2 pigs were selected at random from each group for harvest (TH, TL, DH and DL). These animals were feed-restricted for 12 h and sacrificed by electric shock. Lung samples were harvested immediately and snap frozen in liquid nitrogen and then stored at -80°C until utilized for RNA extraction.

### Total RNA isolation and microarray hybridization

Total RNA was extracted from each lung sample using the Trizol RNA isolation method (Invitrogen, USA). RNA was purified and DNase treated with QIAGEN RNeasy® Mini kit (QIAGEN, Valencia, CA) according to the manufacturer’s instructions. The integrity and quality of the extracted RNA was evaluated by the Agilent 2100 bioanalyzer (Agilent Technologies, Inc., Santa Clara, CA, USA). The RNA Integrity Number (RIN) values of all samples were greater than 8.0. The RNA was amplified and labeled using Agilent Low Input Quik Amp Labeling Kit, One-Color (Agilent Technologies, USA). Labeled cRNA from 8 animals were purified by QIAGEN RNeasy mini kit and hybridized individually (1 sample per array) using commercially available Agilent Whole Porcine Genome Oligo (4 × 44 K) Microarrays (Agilent-020109, one-color platform) according to the instructions in the Agilent Expression Analysis Technical Manual. After the hybridization, the gene chips were washed using Agilent Gene Expression Wash Buffer Kit at room temperature.

### Microarray data analysis and statistics

After drying, slides were scanned using an Agilent G2505B microarray scanner (Agilent Technologies, USA). Image files were analyzed with Agilent Feature Extraction software (version10.7.3.1) to obtain the raw expression data. All raw gene expression values have been deposited in the Gene Expression Omnibus (GEO) database (Accession number: GSE84409). If a transcript was flagged as “A” by the scanner, i.e. signal intensity was less than background level, it was considered to be “not expressed”. Similarly, the transcripts with “P” flags, expression greater than background levels, were considered to be “expressed”. In our study, expressed transcripts were defined as being present in at least one sample. In total, 34,359 expressed probes were used for subsequent analyses, among these, only 16,946 were annotated, corresponding to about 10,000 genes (including LOC). More probes were annotated using GenBank and Basic local Alignment Search tool program (BLAST, NCBI) (http://www.ncbi.nlm.nih.gov/).

Expression data were normalized using Robust Multichip Analysis (RMA). Statistical analysis was performed using two-way ANOVA in an R script developed by Pavlidis et al. [30] to identify differentially expressed genes (DEGs) related to high altitude tolerance. The criterion on detection of DEGs was set at FDR < 0.0001. Gene expression, y = log (expression), was modeled as follows:

$$y_{ijk}=\mu +S_{i}+R_{j}+{\left(S•R\right)}_{ij}+\varepsilon_{ijk}$$

Where *y*_*ijk*_ was dependent variable, *μ* was the mean expression level of the gene, *S*_*i*_ was fixed effect of breed (*i* = 1 to 2), *R*_*j*_ was fixed effect of altitude (*j* = 1 to 2), *(S•R)*_*ij*_ was interaction between breed and altitude, and *Ɛ*_*ijk*_ represented the random error. Thus, the expression level *y* could be impacted by main effects due to *S*, *R*, and interactions between them (*S•R*), plus random error.

### Pathway and functional analysis of DEGs

The probe set IDs for GenBank Accession Number based on the Agilent porcine array annotation file were first mapped to NCBI Entrez gene IDs generated by BioGps software [31]. Gene Ontology and pathway analysis was conducted by PANTHER version 11.1 [32] (http://www.pantherdb.org/) and the online STRING tools (http://string-db.org/) to identify significantly over-represented GO terms, PANTHER (Bonferroni correction *P* < 0.05) and KEGG pathways (FDR<0.01).

### Gene co-expression network construction and RIF analysis

**Fig 2:**
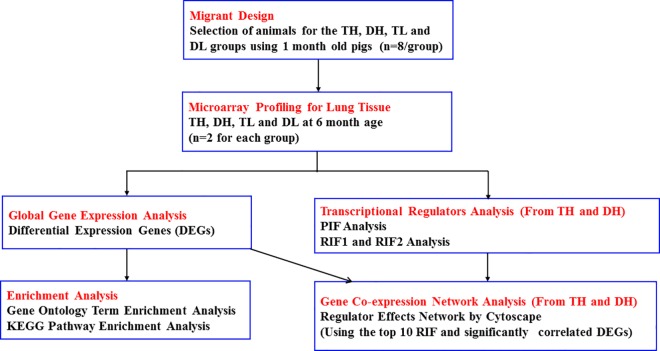
Diagram summarizing experiments and analyses conducted in the whole study. Abbreviations: TH = Tibetan pigs at high altitude, DH = Duroc pigs at high altitude, TL = Tibetan pigs at low altitude, DL = Duroc pigs at low altitude, DEGs = Differential Expression Genes, PCIT = Partial Correlation and Information Theory, RIF = Regulatory Impact Factor, PIF = Phenotype Impact Factor.

A modified version of PCIT [26] was used to compute the co-expression correlation in the TH (Tibetan at high altitude) or DH (Duroc at high altitude) group, respectively. Only transcripts with a significant partial correlation |r| ≥ 0.95 were selected to construct gene co-expression networks. Transcriptional regulators (TRs) include mainly transcription factors, signaling molecules and chromatin remodelers [28]. In this study, these molecules were mined from the literature in human [33] and bovine [28] and from the SWISS-PROT database for swine and mouse (http://www.uniprot.org/) [34]. The final non-redundant transcriptional regulator list of genes in our annotation array totaled 1,897 (S3 Table). According to the RIF and phenotype impact factor (PIF) algorithm [28, 29], the pivotal transcriptional regulators related to adaptation to high altitude can be identified based on the RIF1 or RIF2 rank. Co-expression networks were visualized in Cytoscape v3.2.1 software [35] for TH and DH by selecting the top five positive and negative TRs based on the RIF1 or RIF2 z-scores and their targets (DEGs and differential PIF) with significant partial correlation from PCIT results. The statistical analysis of differential PIF was performed using the same method described for DEGs (two-way ANOVA) and the criterion was set at FDR < 0.0001. All processes, experiments and analyses are shown in Fig 2.

## Results

### Differentially expressed genes

A total of 3,431 differential expression transcripts were identified by two-way ANOVA based on a threshold of FDR < 0.0001, which were represented 3,066 non-redundant genes. These DEGs reflected the difference between breeds (Tibetan vs. Duroc) affected by the interaction that may be responsible for the adaptation of Tibetan pigs to high altitude (S1 and S4 Tables). These genes were significant both due to the interaction effect and also the breed effect. These genes were selected to emphasize the objective to identify genes DE due to adaptation of the breed to differing altitudes.

### Functional analysis of DEGs affected by hypoxia

**Fig 3:**
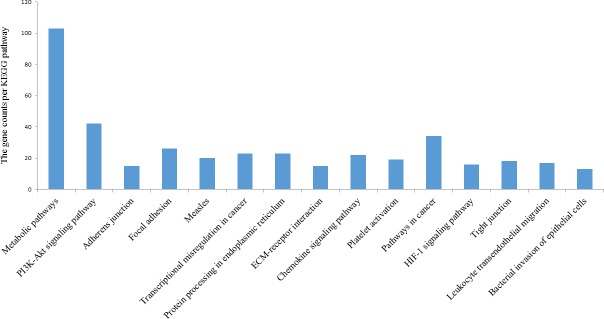
The top 15 enriched KEGG pathway from the list of 3066 differentially expressed genes (DEGs).

The annotations for DEGs were tested for over-representation of biological pathways and processes. Significantly enriched terms included: 7 biological processes (BP), 4 molecular functions (MF), 3 PANTHER pathways, 7 cellular components, 3 PANTHER protein class (Bonferroni corrected *P* **<** 0.05) and 29 KEGG (FDR < 0.01) (S2 Table). The most significantly enriched terms were metabolic process (GO: 0008152), catalytic activity (GO: 0003824) and CCKR signaling map (P06959) in BP, MF and PANTHER pathway (Table 1), respectively. The most significantly enriched KEGG pathways were the metabolic pathway and PI3K-Akt signaling pathway (Fig 3).

**Table 1:** GO terms related to biological processes, molecular function and PANTHER pathways for differentially expressed genes (Bonferroni corrected *P* < 0.05)

**Term**	REF*	**Count**^**^**^	**Expected**^**#**^	**Over/under**^**±**^	**Fold Enrichment**	**Adj.** *P***. value**^**%**^
Biologicalprocess						
GO: 0008152~metabolic process	6594	460	350.07	+	1.31	7.88E-10
GO: 0044238~primary metabolic process	5592	385	296.87	+	1.30	1.01E-06
GO: 0009056~catabolic process	836	74	44.38	+	1.67	4.81E-03
GO: 0019538~protein metabolic process	2001	146	106.23	+	1.37	1.59E-02
GO: 0006807~nitrogen compound metabolic process	1982	151	105.22	+	1.44	1.35E-03
GO: 0040011~locomotion	153	21	8.12	+	2.59	2.59E-02
GO: 0006397~mRNA processing	241	28	12.79	+	2.19	3.46E-02
*Molecular function*						
GO: 0003824~catalytic activity	5114	376	271.5	+	1.38	2.70E-10
GO: 0008009~chemokine activity	46	12	2.44	+	4.91	1.72E-03
GO: 0005515~protein binding	2719	191	144.35	+	1.32	6.75E-03
GO: 0016787~hydrolase activity	2175	153	115.47	+	1.33	4.20E-02
*PANTHER pathway*						
P06959: CCKR signaling map	169	26	8.97	+	2.9	3.85E-04
P00005: Angiogenesis	168	22	8.92	+	2.47	2.24E-02
Wnt signaling pathway (P00057)	278	30	14.76	+	2.03	4.55E-02

Note

*REF: Based on the *S*. *scrofa* reference list for a particular PANTHER category.

^^^Count: number of genes that belong to the given particular category.

^#^Expected: expected value for number of genes that belong to the particular pathway among microarray results.

^±^Over/under: stands for ‘over-represented/under-represented.

^%^Adj. *P*. value: *P*-value corrected by Bonferroni correction for multiple testing.

### Putative transcription regulators of DEGs identified by RIF and PIF

To identify which crucial TRs may affect gene expression difference and above-mentioned GO categories and pathways, we computed PIF, RIF1 and RIF2 scores. All probe’s PIF values are provided in S3 Table. Among these, 3,656 differentially PIF transcripts (FDR < 0.0001) were identified using two-way ANOVA (S4 Table). All RIF scores are provided in S5 Table. We identified 369 non-redundant TRs with RIF z-scores < -2 or > 2. Among them, 117 TRs had RIF1 z-scores > 2 and 101 had RIF1 z-scores < -2. In addition, 136 TRs had RIF2 z-scores > 2 and 125 had RIF2 z-scores < -2. Moreover, 91 TRs were common for RIF1 and RIF2 based on z-scores < -2 or > 2. Tables 2 and 3 listed the top 5 positive or negative extreme regulators, which were selected according to RIF1 or RIF2 z-scores.

**Table 2:** Top 10 differentially wired regulators identified by RIF1 (Tibetan vs Duroc at high altitude).

**Probeset ID**	**Gene Symbol**	**Gene Description**	**RIF1 z-Scores**	**RIF2 z-Scores**
Top positive RIF1				
A _72_P122616	EPC1	enhancer of polycomb homolog 1 (Drosophila)	5.32	1.98
A _72_P067171	LOC100519426	chromobox protein homolog 5	5.10	2.41
A_72_P118106	KLF6	Kruppel-like factor 6	4.87	3.44
A_72_P088426	MAGED1	melanoma antigen family D, 1	4.71	2.49
A_72_P165271	IKBKG	inhibitor of kappa light polypeptide gene enhancer in B-cells, kinase gamma	4.63	2.87
Top negative RIF1				
A_72_P137561	RBPJ	recombination signal binding protein for immunoglobulin kappa J region	-6.53	-3.34
A_72_P042871	POLR2E	polymerase (RNA) II (DNA directed) polypeptide E, 25kDa	-5.63	-2.29
A_72_P036711	TIPARP	TCDD-inducible poly(ADP-ribose) polymerase	-4.22	-3.26
A_72_P098786	WDR5	WD repeat domain 5	-4.17	-2.43
A_72_P100331	SRRT	serrate, RNA effector molecule	-4.12	1.08

**Table 3:** Top 10 regulators identified by RIF2 (Tibetan vs Duroc at high altitude).

**ProbesetID**	**Gene Symbol**	**Gene Description**	**RIF2 z-Scores**	**RIF1 z-Scores**
Top positive RIF2				
A_72_P011236	EFEMP1	EGF containing fibulin-like extracellular matrix protein 1	5.57	1.59
A_72_P011441	HOXB6	homeobox B6	5.37	2.90
A_72_P014051	ATF6	activating transcription factor 6	4.41	2.83
A_72_P024356	ZNF280B	zinc finger protein 280B	4.12	1.09
A_72_P108351	HIST1H3E	histone cluster 1, H3e	3.88	4.27
Top negative RIF2				
A_72_P049681	EPC2	enhancer of polycomb homolog 2 (Drosophila)	-4.74	-1.48
A_72_P013732	ERG	v-ets avian erythroblastosis virus E26 oncogene homolog	-4.62	-1.76
A_72_P008741	HABP4	hyaluronan binding protein 4	-4.13	-1.53
A_72_P030981	ZNHIT6	zinc finger, HIT-type containing 6	-3.93	-2.30
A_72_P006916	SF3B1	splicing factor 3b, subunit 1	-3.77	-1.42

### Co-expression gene patterns at high altitude

**Fig 4:**
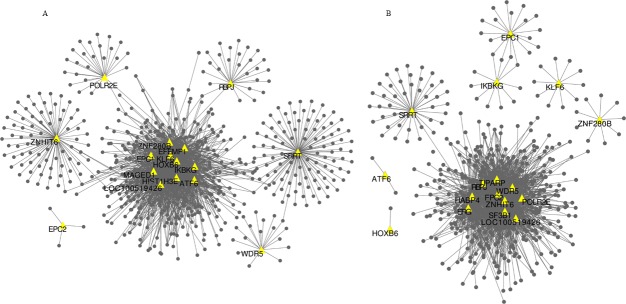
Co-expression networks based on the PCIT method. (A) Tibetan group at high altitude (660 nodes and 1878 edges) (B) Duroc group at high altitude (512 nodes and 1641 edges). Node shape and color were mapped to the different gene types: transcription regulators (triangles, yellow), differential expression genes and differential PIF genes (circles, black).

Here we applied the correlation relationships from the PCIT results to construct co-expression correlation network (|r| ≥ 0.95) between 20 TRs (Tables 2 and 3) with significantly DE and PIF genes in the TH and DH breed groups, respectively. Networks were visualized with Cytoscape software and are presented in (Fig 4). We identified four TRs (*TiPARP*, *SF3B1*, *HABP4*, *ERG*) with no connection to DEGs and PIF genes in the TH group, and three TRs (*EFEMP1*, *MAGED1*, *HIST1H3E*) also with no connection in DH group.

## Discussion

Tibetan pigs have lived at high altitude for thousands of years, which provided an excellent animal model for investigating mechanisms of hypoxia adaptation. Natives of high altitude have been documented to possess genetic variations that may be the results of compensatory genetic changes and natural selection. However, the high altitude environment also plays an important role in adaptation, because environmental effects modify gene transcription and/or translation that may irreversibly affect the phenotype [36]. If we take an integrative approach to analyze the mechanisms related to hypoxia adaptation, the effect of the gene must be evaluated with respect to the way that it interacts with the environment. Therefore, we employed a complete migrant design, where Tibetan pigs and Duroc pigs were raised in both their native altitude environments and also as migrants in a non-native environment. Changes in gene expression due to this migration indicated gene-by-environment interaction [37, 38]. In our study, a total of 3,066 DEGs were identified (FDR < 0.0001) as overlapping due to genetic variance (breed) and genetic×environment interaction effects. These DEGs were significantly over-represented for genes involved in metabolic process, catalytic activity, CCKR signaling and angiogenesis pathway by PANTHER classification categories (S2 Table) (Bonferroni corrected *P* **<** 0.05), and metabolic pathways and the PI3K-Akt signaling pathway by KEGG categories (Fig 3 and S2 Table) (FDR < 0.01).

Gene expression is regulated by TRs that tend not to be differentially expressed, because their activation is often modulated post-transcriptionally, i.e. via reversible phosphorylation, cellular localization, cofactor or ligand binding and so on [39]. To understand the genetic adaptation of Diqing Tibetan pigs to low oxygen, the RIF and PIF algorithms were used to identify potential TRs of DEGs. Correlation of gene expression levels within a network was determined using PCIT to construct regulation and co-expression networks (Fig 4). The PIF value weights the differential expression of a given gene by its overall abundance. The number of differentially PIF genes was greater than the number of DEGs (S4 Table), since differentially PIF genes could either have a large DEG or be a high abundant gene not hugely DEG [29]. RIF1 scores identify potential regulators [29] with large differential wring (i.e. change in correlation) to highly abundant, high differentially expressed genes between the two breeds at high altitude. RIF2 scores rank TRs as potential biomarkers to predict the abundance of DEGs [29]. In our study, we ranked 3,373 TRs (1,897 non-redundant) by RIF1 and RIF2 scores (S5 Table), and the top ten RIF1 or RIF2 TRs were listed in Tables 2 and 3.

In previous studies, the native highlanders were identified to have many adaptive features such as blunted hypoxic pulmonary vasoconstriction, increased erythropoiesis and metabolic reprogramming [5–7]. Moreover, mounting evidence suggests that the observed physiological adaptations are dependent on the HIF pathway [40–42]. In our study, 16 DEGs were also enriched as members of the HIF pathway (Fig 3 and S2 Table). Besides erythropoiesis, additional processes regulated by HIF are angiogenesis, glucose uptake and metabolism, which regulate oxygen delivery and consumption [11, 12, 14, 43]. In our study, most of the TRs identified as putative regulators are involved in response to hypoxia, angiogenesis and metabolic processes. In the top ten RIF1 gene list, inhibitor of kappa light polypeptide gene enhancer in B-cells, kinase gamma (*IKBKG*) gene, Kruppel-like factor 6 (*KLF6*) and recombination signal binding protein for immunoglobulin kappa J region (*RBPJ*) were identified (Table 2). *IKBKG* encodes the inhibitor of the κB kinase gamma (IKKγ) protein, also known as nuclear factor κB (NF-κB) essential modulator (NEMO). IKKγ is essential for the activation of the NF-κB pathway that plays a major role in immune response, inflammation, cell adhesion, cell survival and development [44]. IKKγ also protects HIF2α from proteasomal degradation and promotes its accumulation [45]. Under hypoxic conditions, HIFα forms a heterodimer that stabilizes HIFβ, which binds to hypoxia response elements in the promoter regions of hypoxia sensitive genes such as VEGF to induce gene transcription [46, 47]. HIF-dependent transcriptional changes lead to a broad range of cellular adaptations, including metabolic, proliferative, apoptotic, and angiogenic changes [48, 49]. KLF6 is a ubiquitously expressed member of the Kruppel-like family of transcription factors. KLF6 regulates the expression of multiple genes and is involved in tissue differentiation [50]. Under hypoxia condition, KLF6 is a potential transactivator of inducible nitric oxide synthase expression, which can promote the synthesis of Nitric oxide (NO) [51]. Elevated NO production helps populations living at high altitude avoid hypoxia by promoting vasodilation of pulmonary vasculature [52]. RBPJ is a transcription factor in the Notch signaling pathway [53]. Hypoxia activates the Notch intracellular domains associated with RBPJ recruitment of HIFα as transcriptional coactivators to target Notch downstream genes to regulate sprouting angiogenesis and vascular remodeling [54].

Splicing factor 3b subunit 1 (*SF3B1*), epidermal growth factor containing fibulin-like extracellular matrix protein 1 (EFEMP1), *HOXB6* and activated transcription factor 6 (*ATF6*) were identified as the top-ten ranked RIF2 genes (Table 3). SF3B1 is considered to be a core factor of the spliceosome that contributes to the production of multiple distinct mRNAs [55]. It is identified as a HIFα target. Under hypoxia, the HIFα dependency of induction of a SF3B1 signal pathway enhances activation of fructose metabolism. Fructose metabolism is a central component of hypoxia-driven metabolic programs to maintain macromolecular such as lipids, proteins and nucleotide biosynthetic capacity to support the anabolic energy requirements [56]. EFEMP1 is a member of the fibulin family of extracellular glycoproteins. Fibulins have been shown to modulate cell morphology, growth, adhesion and motility [57]. EFEMP1 expression was positively correlated with microvascular density and the expression of VEGF [58]. HOXB6 is considered as a hallmark of erythropoiesis. Homeobox genes appear to be strong candidate genes to regulate hematopoiesis [59, 60]. The regulation of erythropoiesis is crucial for optimal oxygen delivery to the tissues under hypoxia [61]. ATF6 is an endoplasmic reticulum (ER) stress sensors. “ER stress” can be caused by a multitude of factors, including severe hypoxia [62]. ATF6 modulates ER function to protect cells against chronic stress [63]. Hypoxia induced changes in gene expression likely occur beyond the change of nucleotide sequence through genome-wide alterations in histone modification and DNA methylation [64]. Such modifications may result in global modulation of transcription levels [65]. In the top ten RIF1 or RIF2 genes, WD repeat domain 5 (*WDR5*), histone cluster 1, H3e (*HIST1H3E*) and chromobox protein homolog 5 (*LOC100519426*), were identified as putative regulators. These proteins are associated with histone modifications [66–68]. This result may indicate the potential for epigenetic regulation of adaptation to hypoxia; however, further investigation of offspring in hypoxic environments is needed to determine if this is plausible.

## Conclusions

In the present study, we used a complete migrant design to study the interaction between genetic and environmental factors involved in the adaptation of Tibetan pigs to hypoxia. DEGs were identified due to breed by interaction effects that enriched for genes involved in metabolic processes and pathway, PI3K-Akt signaling pathway and HIF signal pathway. These biological processes could have been directly or indirectly regulated by the partial pressure of oxygen from the environment. In addition, putative pathway regulators of hypoxia adaptation were identified by RIF and co-expression methods. These regulators may affect gene expression by post-transcriptional or epigenetic modification. These findings contribute to a better understanding of the molecular mechanisms potentially underlying hypoxia adaptation, and may provide important information relevant to other species, especially humans.

## Supporting Information

S1 Table Results of microarray data analysis. (XLSX)

S2 Table GO, PANTHER and KEGG pathways results of DEGs. (XLSX)

S3 Table Results of PIF analysis. (XLSX)

S4 Table Differential expression genes and differential PIF. (XLSX)

S5 Table The results of RIF1 and RIF2 z-scores. (XLSX)
